# Advanced photodetector for hybrid PET-MRI systems

**DOI:** 10.12688/openreseurope.18695.1

**Published:** 2024-11-08

**Authors:** K Isayev, O Rasulov, N Sadigova

**Affiliations:** 1 Nuclear Research Department, IDDA, Baku, Azerbaijan; 2 Elmek Mozgasban Alapitvany, Budapest, Hungary; 3 Azerbaijan University of Architecture and Construction, Baku, Azerbaijan

**Keywords:** PET-MRI, SiPM, MAPD, photon detection efficiancy, gain, MAPD-3NK, MAPD-3NM

## Abstract

**Background:**

Currently, a wide variety of silicon photomultipliers (SiPMs) are available, each designed for specific applications in fields such as science, medicine, and industry. Advances in production technology have led to the development of more sensitive and efficient photodiodes, which are critical for applications requiring precision, such as medical imaging.

**Methods:**

A research group has been working on designing a highly sensitive photodiode to enhance the capabilities of next-generation PET-MRI scanners. This involves integrating micropixel avalanche photodiodes (MAPDs) to improve image resolution. The chosen design features deep-immersion MAPDs with a pixel size of 12 microns and a density of 1000 pixels per square millimeter, allowing for high-detail photon detection. The 4x4 mm ^2^ active area is optimized to balance sensitivity and size for high-resolution medical imaging. To produce these photodiodes, the group has outlined a production plan involving 20-inch silicon wafers grown using multiple techniques to enhance material properties. The Malaysian Institute of Microelectronic Systems (MIMOS), renowned for its expertise in optical microelectronics, was selected as the production center. With MIMOS' state-of-the-art facilities, the project aims to meet stringent medical diagnostics standards.

**Results:**

The experimental results demonstrated that the MAPD-3NM photodiode achieved an amplification factor 1.8 times greater than the MAPD-3NK under optimal conditions. Its overvoltage range increased by 100%, reaching 4 V, enhancing photon detection and amplification. The MAPD-3NM also showed a significant reduction in dark current, about 3.5 times lower than the MAPD-3NK, improving performance in low-light environments. Additionally, the MAPD-3NM had a capacitance of 200 pF compared to 176 pF for the MAPD-3NK, contributing to its superior performance. These improvements make the MAPD-3NM more efficient and sensitive for scientific and medical applications.

**Conclusions:**

This project represents a major advancement in photodetector technology for medical diagnostics, aiming to develop more accurate and efficient PET-MRI scanners that enhance patient outcomes with improved imaging capabilities.

## Plain language summary

Oncological diseases are among the most emotionally and mentally taxing medical conditions, and the number of people diagnosed with various types of cancer continues to rise. Early detection is crucial for enabling timely and effective treatment, which requires highly sensitive diagnostic devices. One of the most advanced solutions in this field is the hybrid PET-MRI scanner, offering superior imaging capabilities by combining the metabolic insights from PET scans with the anatomical precision of MRI.

To enhance the performance of these diagnostic systems, our research group has developed highly sensitive avalanche photodiodes with a deep pixel structure. This innovative design enables an impressive pixel density of up to 10,000 pixels per square millimeter. The photodiodes exhibit a photon detection efficiency (PDE) exceeding 30%. These characteristics significantly improve detection sensitivity and image resolution, making the PET-MRI scanner a more powerful tool for early cancer diagnosis and more accurate treatment planning.

By advancing the detection capabilities of hybrid PET-MRI technology, we aim to provide better diagnostic outcomes for patients, allowing for earlier intervention and potentially improved survival rates. Our work underscores the importance of technological innovation in the ongoing fight against cancer, where early and precise detection remains a key factor in successful treatment strategies.

## Introduction

Modern diagnostic equipment represents a remarkable convergence of science and technology, playing a crucial role in improving healthcare outcomes. These devices are not only highly sophisticated but are constantly evolving, as advancements in medical technology allow for earlier and more accurate diagnosis of life-threatening conditions. Early diagnosis is critical because it increases the likelihood of successful treatment, improving survival rates for conditions like cancer and cardiovascular diseases. Among the most innovative diagnostic tools available today is the hybrid PET/MRI scanner ^1^, a device that combines the strengths of positron emission tomography (PET) and magnetic resonance imaging (MRI) to provide comprehensive, simultaneous imaging.

PET and MRI are both powerful imaging modalities, but they operate based on fundamentally different physical principles and deliver unique types of information. MRI is based on the nuclear magnetic resonance phenomenon ^2^, primarily focusing on the behavior of hydrogen atoms. Since the human body is predominantly composed of water molecules, which contain hydrogen atoms, MRI is particularly effective for imaging soft tissues. When the body is placed in a strong magnetic field, the hydrogen nuclei (protons) align along the direction of the magnetic field. A radiofrequency pulse then temporarily displaces these protons, and as they return to their original alignment, they emit signals that can be detected by the MRI scanner. These signals are processed to create highly detailed, cross-sectional images of the body's internal structures. MRI is especially valued for its ability to produce high-contrast images, making it ideal for detecting tumors, brain abnormalities, spinal cord injuries, and other soft tissue conditions.

In contrast, PET focuses on metabolic processes rather than structural details ^3^. PET imaging involves introducing a small amount of a radioactive tracer, typically a radionuclide, into the patient’s body. These radionuclides emit positrons, the antimatter counterparts of electrons. When a positron encounters an electron, the two particles annihilate each other, releasing gamma rays in the process. PET detectors capture these gamma rays, enabling the scanner to visualize metabolic activity. PET is particularly useful in detecting cancers, as cancer cells tend to have higher metabolic rates than normal cells, allowing PET to identify abnormal tissue growths at a cellular level. While PET may not provide the high-resolution structural images that MRI does, it excels in showing how organs and tissues are functioning in real-time. This makes it an invaluable tool for oncology, where it can track the spread of cancer metastases, monitor treatment response, and help plan therapies.

Despite their individual strengths, both PET and MRI have limitations. PET involves exposure to ionizing radiation from the injected radionuclides, which, although minimal, still poses some risk to patients, especially with repeated exposure. MRI, on the other hand, while not involving radiation, has its own risks and limitations, particularly in patients with metal implants or those who are sensitive to the effects of strong magnetic fields. The development of hybrid PET/MRI systems addresses these challenges by combining the functional imaging capabilities of PET with the structural imaging strengths of MRI in a single device. This integration not only improves diagnostic accuracy but also enhances patient safety by reducing the overall radiation exposure compared to standalone PET or PET/CT systems.

The hybrid PET/MRI system introduces a new era in medical imaging by allowing physicians to conduct simultaneous functional and structural studies. The device’s high sensitivity, particularly due to advancements in the photodetector technology it employs, allows for a significant reduction in the dose of radiopharmaceuticals administered to patients. This ensures that diagnostic procedures remain highly accurate while minimizing radiation exposure. For example, in brain research, PET/MRI enables simultaneous mapping of brain function and real-time observation of radiopharmaceutical uptake, offering unique insights into the progression of neurodegenerative diseases such as Alzheimer’s, epilepsy, and Parkinson’s disease. This capability is critical for both diagnosis and monitoring of treatment efficacy, as functional changes in the brain can be tracked alongside anatomical alterations.

The hybrid PET/MRI system also holds great promise in the field of oncology. In cancers such as those of the prostate, breast, cervix, brain, and liver, the device can provide precise localization of tumors, assess the stage of disease, and evaluate the patient’s response to treatment. One notable advantage of PET/MRI over PET/CT is its superior soft tissue contrast, which is particularly important in detecting tumors in areas like the brain or liver, where tissue differentiation is crucial. Moreover, PET/MRI reduces radiation exposure by eliminating the need for the computed tomography (CT) scan, making it a safer alternative, especially for pediatric patients and those requiring multiple scans over time.

Another area where PET/MRI shows significant potential is in the diagnosis and management of inflammatory diseases, such as Crohn's disease. Crohn's disease is a chronic inflammatory condition of the gastrointestinal tract that can be difficult to diagnose, particularly in its early stages. The hybrid PET/MRI system enables early detection by combining PET's ability to detect metabolic changes associated with inflammation and MRI's ability to provide detailed anatomical images of the gastrointestinal tract. This combination can help physicians identify and monitor the disease before it causes significant damage to the digestive system.

Cardiovascular imaging is yet another promising application of PET/MRI technology. The system offers a unique capability to assess coronary blood flow and myocardial viability. MRI allows for detailed visualization of the heart, enabling doctors to monitor its structure, function, and dynamics, such as the contraction of the heart chambers and the thickness of the myocardium. PET, on the other hand, is invaluable in detecting metabolic activity in the myocardium, allowing for the identification of viable heart tissue in areas affected by conditions such as ischemia or myocardial infarction. The ability to detect living tissue within scarred areas, for example after an ischemic heart attack, could significantly improve the diagnosis and treatment planning for patients with heart disease.

The potential for PET/MRI in pediatric care is also noteworthy. Children are particularly sensitive to radiation, and minimizing their exposure is a key concern in medical imaging. The hybrid PET/MRI system offers a significant advantage in this regard by reducing radiation doses compared to traditional PET/CT or standalone PET scans. This makes PET/MRI a safer option for pediatric patients, who may require repeated imaging over the course of their treatment.

In line with the World Health Organization’s (WHO) Cancer Control Planning guidelines, which emphasize the importance of early detection, diagnosis, treatment, and palliative care, hybrid PET/MRI systems are poised to play a critical role in improving cancer outcomes. By leveraging the latest advances in digital technology and photodetector design, these systems offer the potential to enhance diagnostic accuracy while reducing the risks associated with radiation exposure. The WHO has identified five key action areas where member states can contribute to cancer control: prevention, early detection, diagnosis and treatment, palliative care, and policy development. Within these areas, the development of innovative diagnostic tools like PET/MRI will be essential for achieving the goals of improved early detection and more effective treatment strategies.

In recent years, significant efforts have been made to improve the performance of silicon photodetectors ^4– 8^. Silicon photodetectors are critical components in detecting the gamma radiation emitted during PET imaging, and their performance directly impacts the quality and resolution of the images produced. However, commercially available designs have limitations, particularly regarding the geometric efficiency of the active surface, which results in reduced photon detection efficiency. To address this, researchers have developed a new design for micropixel avalanche photodiodes (MAPD-3NM). These photodiodes have been applied in a range of fields, including gamma spectroscopy, time-of-flight experiments, alpha particle detection, LIDAR systems, and nuclear forensics, demonstrating their versatility and effectiveness. The MAPD-3NM design offers improved geometric efficiency and photon detection capability, making it a promising solution for enhancing the performance of PET/MRI scanners.

Overall, the continued advancement of hybrid PET/MRI systems, supported by improvements in photodetector technology, represents a significant leap forward in medical imaging. By offering a safer, more accurate, and more comprehensive diagnostic tool, these systems are poised to transform the landscape of medical diagnostics, particularly in oncology, neurology, cardiology, and pediatric care.

## Structure of micropixel avalanche photodiodes

Currently, silicon avalanche photodiodes with surface-pixel architectures are widely implemented in various applications. In these designs, the individual pixels are arranged on the surface of the diode, with each pixel connected to the cathode through a quenching resistor, forming a parallel circuit where isolation channels separate each pixel. However, a portion of the sensitive area is occupied by quenching resistors, isolation channels, and main signal lines, reducing the geometric efficiency of the photodiode and ultimately limiting its photon detection efficiency (PDE). As the pixel density increases in such surface-pixel structures, this geometric factor diminishes sharply, leading to a substantial decline in the device's overall detection efficiency.

An example of this limitation is evident in MPPC S12572-010P photodiodes from HAMAMATSU PHOTONICS™ (Japan), which have pixel density 10,000 pixels/mm ^2^, with a photon detection efficiency of 10%. These constraints hinder the effectiveness of surface-pixel photodiodes for use in advanced applications, such as in PET-MRI scanners, where high efficiency is crucial for accurate medical imaging.

To address these limitations, our research group proposed a novel deep-pixel architecture for silicon avalanche photodiodes ^9, 10^. Unlike the surface-pixel design, in which the pixels are confined to the surface, deep-pixel photodiodes embed each pixel within the epitaxial layer of the silicon. In this structure, each pixel connects to the substrate via the first epitaxial layer, while the potential barrier between the pixel and the epitaxial layer serves the role of the quenching resistance. This approach minimizes the space required for quenching resistors and isolation channels, significantly improving both the geometric factor and photon detection efficiency.

**Figure 1.**
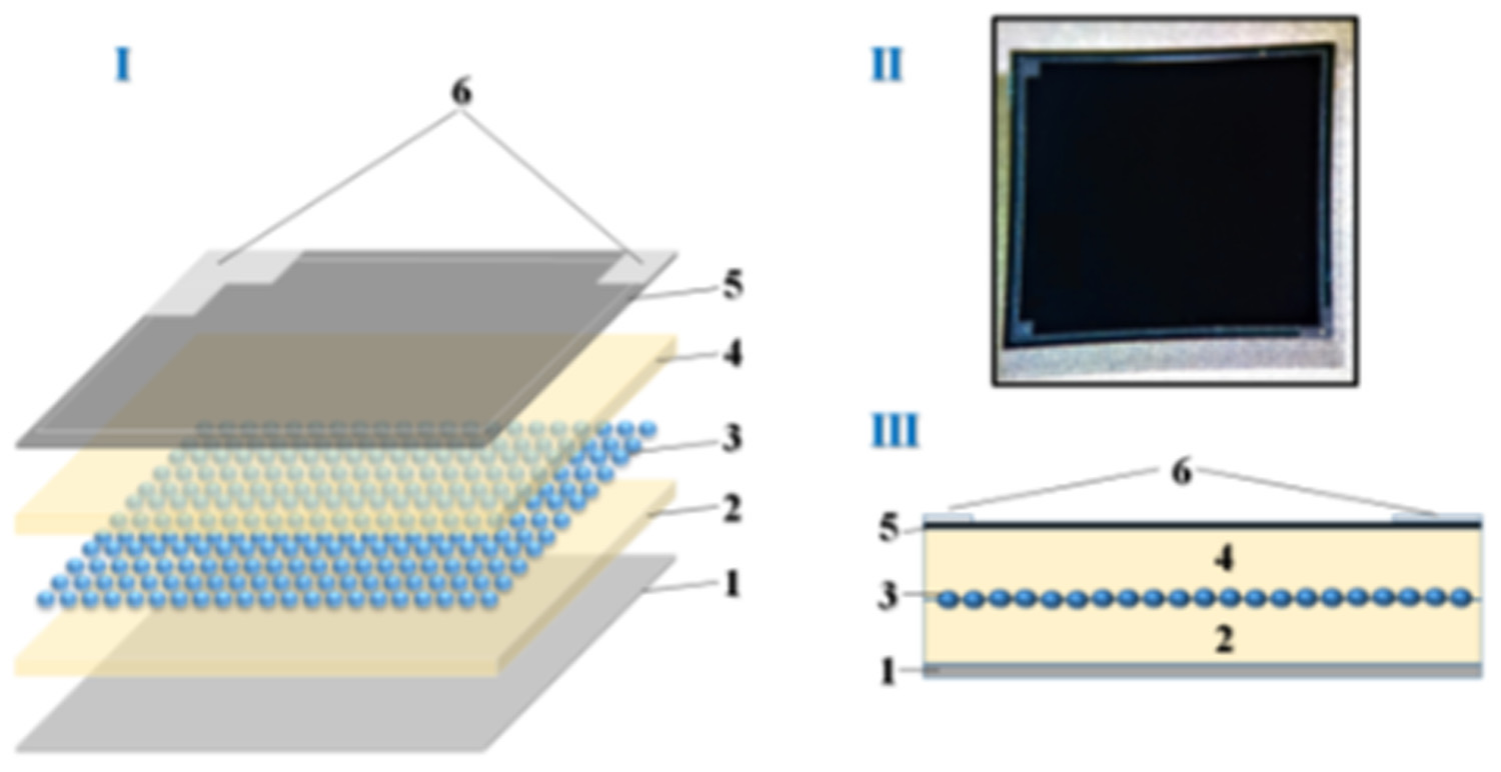
I- Deep pixel photodiode structural scheme, II- photo, III- cross-section. I Layer-by-layer diagram of the photodiode itself consisting of 1- silicon substrate, 2- lower epitaxial layer, 3- pixel matrix, 4- upper epitaxial layer, 5- protective layer - silicon nitride and 6- contact strip. II-real photo. III-Cross-section of the layered structure according to I.

Deep-pixel avalanche photodiodes ( Figure 1), commonly referred to as micropixel avalanche photodiodes (MAPDs), were first introduced by Zecotek Photonics Inc. in 2009 ^11^. Since then, MAPDs have undergone extensive development, with different variations designed for specific applications. The MAPD-3B series, for instance, was the first to be designed with a pixel density of 40,000 pixels/mm ^2^, featuring a pixel size of 2 μm and a pitch of 5 μm. These photodiodes were optimized for high-energy physics experiments, yet they exhibited limitations, including low gain (approximately 10 ^4^) and low photon detection efficiency (~10%).

In an effort to enhance these parameters, the MAPD-3A series was developed with larger pixels (3 μm), reducing the pixel density to 15,000 pixels/mm ^2^, but increasing the gain to ~3×10 ^4^ and improving the registration efficiency to ~15%. However, for applications such as PET-MRI scanners, the photon detection efficiency still fell short of the required 20%.

Further advancements were made with the introduction of the MAPD-3N photodiodes, where the pixel size was increased to 5 μm and the pitch was adjusted to 7 μm. This iteration provided a gain of ~6×10 ^4^ and a PDE of ~25%, with a pixel density of 15,000 pixels/mm ^2^. Despite these improvements, further enhancements were necessary to achieve the desired performance levels for medical imaging applications.

The next generation, the MAPD-3NK photodiodes ^12– 14^, featured a larger pixel diameter (7 μm) and pitch (10 μm), resulting in a pixel density of 10,000 pixels/mm ^2^. Although the MAPD-3NK exhibited a gain of ~6×10 ^4^ and a PDE of ~30%, it also presented several drawbacks, such as a high operating voltage exceeding 90 V, a high temperature coefficient for the breakdown voltage (>50 mV/K), and elevated dark current levels. These shortcomings made MAPD-3NK photodiodes unsuitable for many precision applications.

To address these issues, researchers developed the MAPD-3NM series ^15– 18^ by refining the epitaxial layer structure and doping concentrations. In MAPD-3NM photodiodes, the second epitaxial layer thickness was reduced to 3 μm, and the additive concentration in the first epitaxial layer was increased. These modifications were aimed at lowering the operating voltage, reducing dark current, and improving the temperature stability of the photodiodes.

This paper focuses on the comparative study of the parameters of the MAPD-3NK and the newly developed MAPD-3NM photodiodes, both of which were fabricated by Zecotek. The MAPD-3NK devices were manufactured at the National Nanofab Center (NANOFAB) in South Korea in 2013, while the MAPD-3NM photodiodes were produced by Malaysia’s MIMOS (Malaysia National Application Research and Development Center). Both types of photodiodes share the same pixel diameter and pitch, with a pixel density of 10,000 pixels/mm ^2^.

In the experimental setup, a Keithley 6487 picoammeter/voltage source and an E7-20M "Imitance Meter" were used to characterize the current-voltage and capacitance-voltage characteristics of the photodiodes. These measurements provided insights into the performance improvements brought about by the new epitaxial structure in the MAPD-3NM, as well as its implications for applications in high-precision fields such as PET-MRI scanning, where high PDE and stability under varying operational conditions are critical for effective performance.

The described structure ( Figure 1) is composed of several layers, beginning with a silicon substrate (1). On this substrate, an epitaxial layer (2) is grown, with a thickness that can vary between 3 μm and 7 μm, depending on the specific modification of the device. The pixel matrix (3) is formed through ion implantation within this epitaxial layer, with pixel sizes ranging from 2 μm to 15 μm, depending on the configuration used in different applications.

Following the pixel matrix, a second epitaxial layer (4) is deposited on top, also ranging in thickness from 3 μm to 7 μm, depending on the variant. This layer serves to insulate and protect the pixel matrix, enhancing the photodiode's performance by minimizing noise and optimizing signal integrity. A protective layer of silicon nitride (5) is then applied on top, with a thickness of approximately 100 nm. This silicon nitride layer acts as a passivation layer, shielding the sensitive regions of the device from environmental factors such as moisture and contaminants, thus improving the overall stability and longevity of the photodetector.

Finally, a contact strip (6) is applied to allow electrical connections to be made, ensuring that the photodiode can be effectively integrated into larger electronic systems. The structure’s design, which incorporates both epitaxial layers and protective silicon nitride, provides enhanced stability and improved detection capabilities, making it suitable for high-sensitivity applications such as medical imaging or high-energy physics experiments. This multi-layered approach to photodiode construction is integral to optimizing pixel density, photon detection efficiency (PDE), and minimizing operational drawbacks such as high dark current or excessive voltage requirements.

### Methods

**Figure 2.**
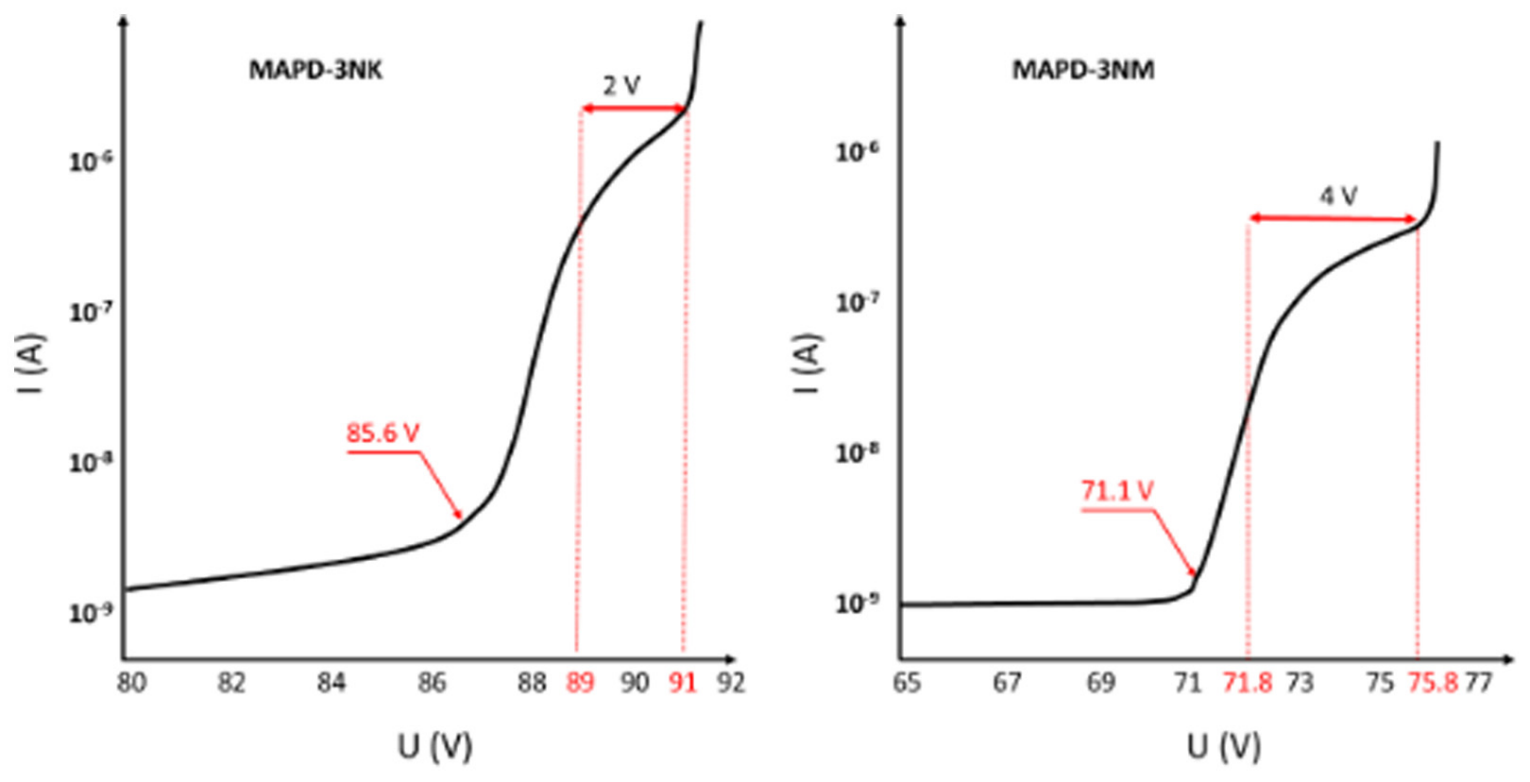
Reverse volt-ampere characteristics of MAPD-3NK and MAPD-3NM photodiode. Voltages of 85.6 V and 71.1 V correspond to the breakdown voltages of the samples, and the range of 89–91 V and 71.8–75.8 V correspond to the working voltage ranges of the samples, respectively.

Figure 2 presents the current-voltage (I-V) characteristics of the MAPD-3NK and MAPD-3NM photodiodes, measured in reverse bias. In both types of photodiodes, the voltage range between 80V and 70.4V corresponds to the low-gain mode (∆U<0V, where ∆U represents the difference between the operating voltage and the breakdown voltage, ∆U = U _operation_ - U _breakdown_). In this low-gain region, the photodiodes exhibit minimal amplification. However, at higher voltages (∆U>0V), the gain increases significantly, entering what is known as the Geiger mode, where the photodiodes operate at peak efficiency.

For the MAPD-3NK photodiode, the voltage range where this high-gain behavior occurs is between 89V and 91V, while for the MAPD-3NM photodiode, this range is much lower, between 71.8V and 75.8V. This indicates that the MAPD-3NM photodiode can achieve high gain at considerably lower operating voltages compared to the MAPD-3NK model, which is a key improvement in terms of energy efficiency and performance optimization.

Another notable difference between the two devices is their dark current, which refers to the current that flows through the photodiode in the absence of light. At their respective operating voltages, the MAPD-3NK photodiode exhibits a dark current of 1485 nA, whereas the MAPD-3NM photodiode shows a significantly lower dark current of 420 nA. This reduction in dark current in the MAPD-3NM photodiode can be attributed to improvements in its epitaxial layer design and overall structural enhancements, which help minimize noise and improve the signal-to-noise ratio in sensitive applications.

These findings highlight the superior performance of the MAPD-3NM photodiode in terms of both lower operating voltage and reduced dark current, making it a more suitable candidate for high-precision applications such as PET-MRI scanners, where high gain, low noise, and efficient energy consumption are critical.

**Figure 3.**
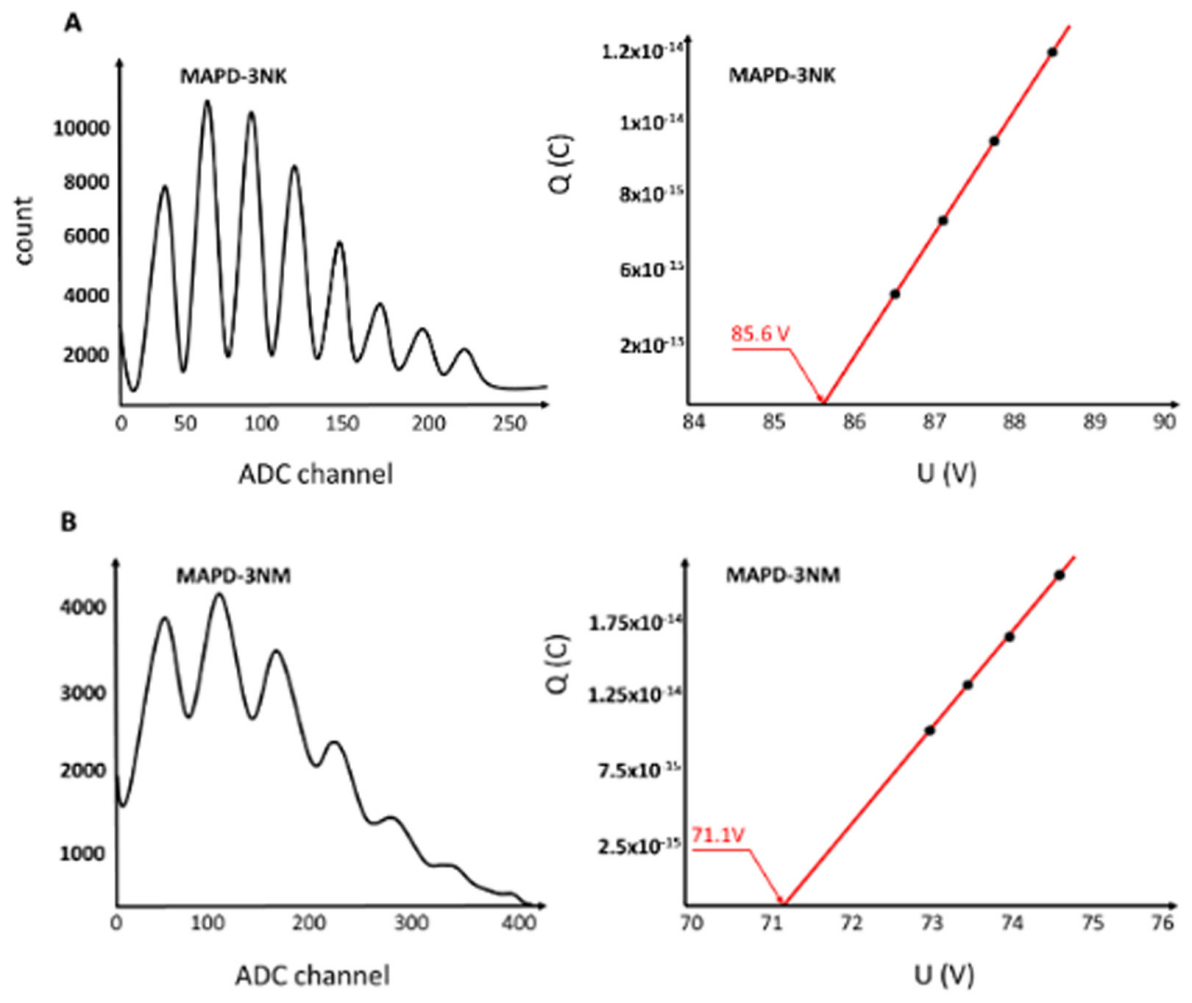
Amplitude distribution of single photoelectrons in MAPD-3NK and MAPD-3NM photodiodes and voltage dependence of the charge corresponding to the first photoelectron. The distance in ADC channels between two successive photoelectron peaks represents the charge of 1 photoelectron (Peaks from left to right correspond to the 1st, 2nd, 3rd......etc. photoelectron). **3A**: the MAPD-3NK photodiode's effective performance under low-light conditions. **3B**: MAPD-3NM photodiodes demonstrate a breakdown voltage of 71.8V.

Figure 3 illustrates the spectral distribution of single photoelectrons generated in response to a weak light beam incident on the photodiodes. The MAPD-3NK ( Figure 3A) photodiode’s breakdown voltage is 85.6 V, which signifies the point at which the photodiode can no longer operate in its linear range and begins to conduct significant current due to avalanche multiplication. At the optimal operational voltage of 90 V, the gain achieved by these photodiodes is approximately 6×10 ^4^ at a temperature of 20 °C. This high gain indicates the device's ability to effectively amplify the signals produced by incoming photons, making it well suited for applications that require high sensitivity.

Furthermore, the maximum overvoltage applied to the MAPD-3NK photodiodes is to be 2V. Overvoltage refers to the voltage difference between the operating voltage and the breakdown voltage, which plays a crucial role in enhancing the gain and improving the overall performance of the photodiode by facilitating a more significant avalanche effect.

Additionally, each pixel of the MAPD-3NK photodiode has a capacitance of 4.2 fF. This capacitance value is important because it reflects the ability of each pixel to store charge, which directly affects the speed of the photodiode's response to incoming photons. A lower capacitance is generally desirable in high-speed applications, as it allows for quicker discharge and faster signal processing.

Overall, the results presented in Figure 3(A) highlight the MAPD-3NK photodiode's effective performance under low-light conditions, characterized by a favorable combination of breakdown voltage, gain, overvoltage, and pixel capacitance. These parameters collectively enhance the photodiode's capabilities, particularly in fields requiring precise light detection, such as medical imaging or particle physics. The ability to achieve high gain at a relatively low operating voltage, combined with low capacitance, positions the MAPD-3NK as a promising candidate for advanced photodetection applications.

The MAPD-3NM photodiodes demonstrate a breakdown voltage of 71.8V. When operated at an optimal voltage of 74.8 V, these photodiodes achieve a gain of approximately 1.1×10 ^5^ at a temperature of 20 °C, as illustrated in Figure 3(B). This elevated gain signifies the device's enhanced sensitivity to low light levels, making it particularly effective for applications that require the detection of weak optical signals.

Additionally, the maximum overvoltage applied to the MAPD-3NM photodiodes is reported to be 4 V. This overvoltage, which is the difference between the optimal operating voltage and the breakdown voltage, is critical for enhancing the gain by promoting greater avalanche multiplication within the photodiode. The ability to achieve a higher overvoltage contributes to the device's improved performance in photodetection applications.

Furthermore, the capacitance of each pixel in the MAPD-3NM photodiodes is measured at 5.7 fF. This capacitance level indicates the pixel's ability to store charge, influencing the speed at which the photodiode can respond to incoming light. Lower capacitance values are generally favorable in fast-response scenarios, as they facilitate quicker discharge and enhance the overall detection rate.

Overall, the advancements presented in the MAPD-3NM photodiodes demonstrate an improvement in the overvoltage range of approximately 100% compared to previous models. This enhancement not only increases the gain and overall sensitivity of the device but also positions the MAPD-3NM as a robust option for high-performance applications in fields such as medical diagnostics and high-energy physics. The combination of a lower breakdown voltage, higher gain, increased overvoltage, and slightly higher pixel capacitance suggests that the MAPD-3NM photodiodes represent a significant step forward in photodetector technology, optimizing performance in low-light environments while ensuring efficient operation.

**Figure 4.**
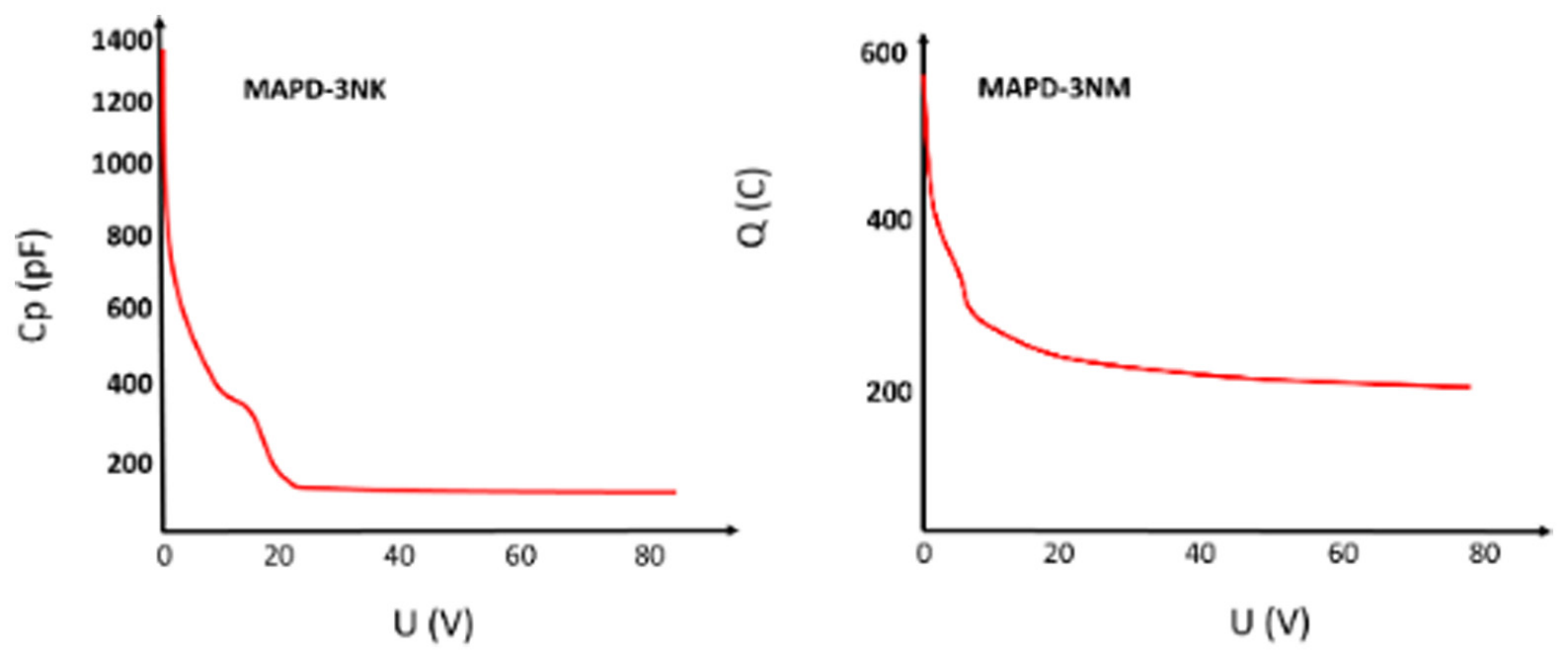
Voltage dependence of MAPD-3NK and MAPD-3NM photodiode capacitance. Measured terminal capacitance of the device under different bias.

The capacitance measurements ( Figure 4) of MAPD-3NK and MAPD-3NM photodiodes reveal distinct electrical behaviors influenced by the structure of their epitaxial layers. For the MAPD-3NK photodiode, at 23 V, the depletion region fully envelops the epitaxial layer, reducing the capacitance, which stabilizes at 176 pF as the voltage increases beyond 24 V. In contrast, the MAPD-3NM photodiode shows a more stable capacitance behavior, reaching 200 pF when the voltage exceeds 10 V, due to its thinner epitaxial layer allowing for greater charge accumulation. These differences in capacitance highlight the impact of epitaxial layer thickness on performance, with MAPD-3NK favoring faster response times and lower noise, and MAPD-3NM providing higher charge-handling capacity and greater sensitivity, making each suited for different high-sensitivity photodetection applications.

## Results

The experimental results revealed that the MAPD-3NM photodiode achieved an amplification factor 1.8 times greater than that of the MAPD-3NK photodiode under optimal conditions. Additionally, the overvoltage range for the MAPD-3NM was increased by approximately 100%, reaching a maximum value of 4 V. This increase in overvoltage significantly enhanced the photodiode's performance by improving its ability to detect and amplify incoming photons.

One of the key improvements observed in the MAPD-3NM photodiode was the significant reduction in dark current, which was ~3.5 times lower than that of the MAPD-3NK photodiode at the operating voltage. Dark current, which represents the unwanted current that flows through a photodiode in the absence of light, is a critical factor in determining the overall noise and sensitivity of the device. The reduction in dark current in the MAPD-3NM photodiode suggests that it offers better performance in low-light environments, enhancing the signal-to-noise ratio and making it more effective for precise photodetection.

Furthermore, the capacitance of the MAPD-3NM photodiode was measured to be 200 pF, whereas the MAPD-3NK photodiode exhibited a capacitance of 176 pF. The higher capacitance in the MAPD-3NM photodiode could be indicative of its increased ability to store charge, which, when combined with its other improved parameters, contributes to its superior performance in various experimental applications.

The results indicate that the newly developed MAPD-3NM photodiode outperforms the MAPD-3NK photodiode across several key parameters, suggesting its potential for broader use in scientific and medical experiments. The enhanced amplification factor, expanded overvoltage range, and significantly reduced dark current make the MAPD-3NM a more efficient and sensitive option for applications requiring accurate photon detection.

Moreover, future developments for the MAPD-3NM series aim to further enhance its performance by focusing on the following improvements:

• **Reduction in dark current**: By improving the quality of the epitaxial layer and reducing the wafer thickness, the dark current can be further minimized, leading to even lower noise and improved sensitivity.

• **Lowering operating voltage**: By adjusting the thickness of the epitaxial layer and optimizing the concentration of additives, it is expected that the operating voltage can be reduced to a range of 50-60 V, making the device more energy-efficient.

• **Increasing the amplification factor**: By increasing pixel capacity and optimizing the overvoltage, the amplification factor can be further enhanced, providing better signal amplification for photon detection.

• **Improving photon detection efficiency (PDE)**: By increasing both the overvoltage and the pixel area, the photon detection efficiency can be boosted, enabling more accurate and efficient detection of light across various experimental setups.

These planned enhancements signal the potential for the MAPD-3NM photodiodes to set new benchmarks in performance, positioning them as highly effective tools for advanced photodetection in areas such as medical imaging, nuclear physics, and other scientific research.

## Data Availability

Open Science Framework: Advanced photodetector for hybrid PET-MRI systems, https://doi.org/10.17605/OSF.IO/5ZGU2 Open Science Framework: Advanced photodetector for hybrid PET-MRI systems https://doi.org/10.17605/OSF.IO/9GJZK This project contains the following data: MAPD 3 NK Breakdown voltage.xlsx (breakdown measurement data of MAPD3NK photodiode) MAPD 3 NK capacitance (capacitance measurement data of MAPD3NK photodiode) MAPD 3 NK spectrum (spectrum performance data of MAPD3NK photodiode) MAPD 3 NK Volt-Ampere (Volt-Ampere characteristic data of MAPD3NK photodiode) MAPD 3 NM Breakdown voltage.xlsx (breakdown measurement data of MAPD3NM photodiode) MAPD 3 NM capacitance (capacitance measurement data of MAPD3NM photodiode) MAPD 3 NM spectrum (spectrum performance data of MAPD3NM photodiode) MAPD 3 NM Volt-Ampere (Volt-Ampere characteristic data of MAPD3NM photodiode) Table of Abbreviations Data are available under the terms of the Creative Commons Zero "No rights reserved" data waiver (CC0 1.0 Public domain dedication) ( http://creativecommons.org/publicdomain/zero/1.0/). Open Science Framework: Advanced photodetector for hybrid PET-MRI systems https://doi.org/10.17605/OSF.IO/9GJZK This project contains the following data: images (folder containing the set of images used for the data analysis) Data are available under the terms of the Creative Commons Zero "No rights reserved" data waiver (CC0 1.0 Public domain dedication) ( http://creativecommons.org/publicdomain/zero/1.0/).
