# Supplementary material for: Advanced photodetector for hybrid PET-MRI systems
Source: Open Res Eur. 2025 Mar 31;4:247. Originally published 2024 Nov 8. [Version 2] doi: 10.12688/openreseurope.18695.2 (PMC11973626; doi:10.12688/openreseurope.18695.2)
Supplement: Peer review history

# Reviewer report: Reviewer response for version 2

**DOI:** 10.21956/openreseurope.21293.r52644

**Published:** 2025-04-03

**Authors:** Gadir Ahmadov

**Affiliations:** 1 Joint Institute for Nuclear Research Joliot-Curie 6, Dubna, Russian Federation

The article presents an insightful analysis of advancements in silicon photomultipliers (SiPMs), specifically focusing on the development of highly sensitive micropixel avalanche photodiodes (MAPDs) for hybrid positron emission tomography (PET) and magnetic resonance imaging (MRI) scanners. The study highlights the importance of improving photon detection efficiency and reducing noise to enhance medical imaging capabilities.

 The authors present a comparative analysis of the MAPD-3NM photodiodes in contrast to the previous MAPD-3NK type, discussing key improvements in parameters such as gain, overvoltage, photon detection efficiency, and dark current. The study effectively demonstrates significant enhancements in photodiode performance, supported by rigorous experimental data.

 One of the primary strengths of this research is the integration of MAPDs into PET-MRI scanners, which represents a crucial advancement in medical imaging technology. This development has the potential to facilitate earlier and more accurate cancer diagnoses, improving patient outcomes.

 Additionally, the research methodology is well-structured, providing a detailed account of the fabrication process, experimental setup, and performance comparisons. This clarity enhances the reproducibility of the study, allowing other researchers to validate or build upon the findings. Furthermore, the discussion on future improvements—including reducing dark current, optimizing operating voltage, and enhancing photon detection efficiency—further reinforces impact and relevance of the article.

 Therefore, this article is highly recommended for researchers, medical physicists, and engineers specializing in medical imaging, nuclear physics, and optical fields. In its current form, it is well-suited for publication. I recommend it for indexing.

Is the study design appropriate and does the work have academic merit?

Yes

Is the work clearly and accurately presented and does it cite the current literature?

Yes

If applicable, is the statistical analysis and its interpretation appropriate?

Yes

Are all the source data underlying the results available to ensure full reproducibility?

Yes

Are the conclusions drawn adequately supported by the results?

Yes

Are sufficient details of methods and analysis provided to allow replication by others?

Yes

Reviewer Expertise:

I am a specialist in the field of silicon photomultipliers and detectors.

I confirm that I have read this submission and believe that I have an appropriate level of expertise to confirm that it is of an acceptable scientific standard.

# Reviewer report: Reviewer response for version 2

**DOI:** 10.21956/openreseurope.21293.r52570

**Published:** 2025-04-02

**Authors:** Christos M. Michail

**Affiliations:** 1 University of West Attica, Athens, Greece

My previous comments were addressed.

Is the study design appropriate and does the work have academic merit?

Yes

Is the work clearly and accurately presented and does it cite the current literature?

Yes

If applicable, is the statistical analysis and its interpretation appropriate?

Not applicable

Are all the source data underlying the results available to ensure full reproducibility?

Yes

Are the conclusions drawn adequately supported by the results?

Yes

Are sufficient details of methods and analysis provided to allow replication by others?

Yes

Reviewer Expertise:

Medical detectors

I confirm that I have read this submission and believe that I have an appropriate level of expertise to confirm that it is of an acceptable scientific standard.

# Reviewer report: Reviewer response for version 2

**DOI:** 10.21956/openreseurope.21293.r52569

**Published:** 2025-04-02

**Authors:** Andrea Gonzalez-Montoro

**Affiliations:** 1 Instituto de Instrumentación para Imagen Molecular, I3M-CSIC-UPV, Valencia, Spain

Dear Editor and authors,

 Thanks for considering my comments and suggestions. I consider you have addressed all my requests and clarified my questions/concerns.

 I believe this is an interesting article for the community and thus, I endorse the acceptance of your work.

Is the study design appropriate and does the work have academic merit?

Yes

Is the work clearly and accurately presented and does it cite the current literature?

Partly

If applicable, is the statistical analysis and its interpretation appropriate?

Not applicable

Are all the source data underlying the results available to ensure full reproducibility?

Yes

Are the conclusions drawn adequately supported by the results?

Yes

Are sufficient details of methods and analysis provided to allow replication by others?

Partly

Reviewer Expertise:

PET instrumentation; SiPM technology;

I confirm that I have read this submission and believe that I have an appropriate level of expertise to confirm that it is of an acceptable scientific standard.

# Reviewer report: Reviewer response for version 1

**DOI:** 10.21956/openreseurope.20225.r46084

**Published:** 2024-11-25

**Authors:** Christos M. Michail

**Affiliations:** 1 University of West Attica, Athens, Greece

The study reports on the technological improvement of the MAPD-3NM integrating micropixel avalanche photodiode, for hybrid PET/MRI applications. Data are presented in comparison with the previously developed MAPD-3NK. The methods are well described and the results are presented in a clear way. The figures are of low resolution, and in some cases the axes are not readable. Since the manuscript refer to combined PET/MRI medical imaging systems, a discussion could be included regarding the operation of the photodiodes in high magnetic fields, to support PET/MR imaging compatibility with the MRI unit. The cited literature can be enriched, especially for the various unsupported statements in the introduction section.

**Specific comments**

**Introduction**

 ‘To address this, researchers have developed a new design for micropixel avalanche photodiodes (MAPD-3NM).’ Please specify and provide appropriate references.

 ‘a larger pixel diameter’ Please keep the terminology consistent for the various terms across manuscript.

**Methods**

 ‘overall structural enhancements’ Please provide some more detailed information.

Is the study design appropriate and does the work have academic merit?

Yes

Is the work clearly and accurately presented and does it cite the current literature?

Yes

If applicable, is the statistical analysis and its interpretation appropriate?

Not applicable

Are all the source data underlying the results available to ensure full reproducibility?

Yes

Are the conclusions drawn adequately supported by the results?

Yes

Are sufficient details of methods and analysis provided to allow replication by others?

Yes

Reviewer Expertise:

Medical detectors

I confirm that I have read this submission and believe that I have an appropriate level of expertise to confirm that it is of an acceptable scientific standard.

# Reviewer report: Reviewer response for version 1

**DOI:** 10.21956/openreseurope.20225.r46089

**Published:** 2024-11-21

**Authors:** Andrea Gonzalez-Montoro

**Affiliations:** 1 Instituto de Instrumentación para Imagen Molecular, I3M-CSIC-UPV, Valencia, Spain

In the present manuscript the authors described the main performance parameters of their newly developed MAPD-3NM devices. They also provide a comparative study of these devices with the previous version (MAPD-3NK) and highlight its superior performance for high precision applications such as PET-MRI.

 I believe this is an interesting work but requires some amendments. I would consider the indexing of the present manuscript after carefully addressing the comments hereafter.

 General: Grammar and explanations are clear and appropriate. But some more references are needed.

 In the following: MA=Major comment, MI = Minor comment, OP = Optional Comment

 (MI) P1, Methods: PET and MRI have not been introduced. Include their meaning before using them.

 (OP) P1, Methods: Instead of writing “per square millimeter” write “mm^2”

 You are using both the imperial (ex: inches) and metric (ex: square millimeter) systems. Try to use only one.

 (MI) P2, Results: Explain what does it mean 3NM and 3NK. This is not clear for non-experts and thus, cannot understand the comparison.

 (MI) P2, Plain Language Summary: It seems that the author claimed in this sentence “The photodiodes exhibit a photon detection efficiency (PDE) exceeding 30%.” that their new devices present better PDE than previous models. If this is the case, please, rewrite. There are SiPM devices with higher PDE being already used.

 (MI) P2, Keywords: there is a typo in “efficiancy” should be “efficiency”

 (MA) P4, Introduction: The authors need to provide more references. In particular the ones regarding the fundaments of PET and MRI, and the relevance in the mentioned diseases and conditions.

 (MI) P4, Introduction: Regarding PET, reading the first paragraphs it seems that PET is only use in oncology (despite in the second part of the introduction it is mentioned the other applications). Please, clarify that PET has more applications in the first paragraphs as well.

 (MA) P4, Introduction: I do not think that PET dosage is significantly reduced when combining PET/MRI. Check this and if this is correct add references. Also, provide some number regarding the dose reduction of PET/CT vs PET/MRI.

 (OP) P4, Introduction: Regarding PET/MRI vs PET/CT, which techniques is more often used and why? And also, what is the future for PET/MRI?

 (MI) P5, Introduction: Add references for the SiPM section. It would be useful for the author if you provide some numbers on the actual performance of state-of-the-art SiPMs.

 (MI) P6, Structure of micropixel avalanche photodiodes: enhance the resolution of Figure 1, it looks blurry. Add dimensions if possible.

 (MI) P6, Structure of micropixel avalanche photodiodes: What is the PDE of the MAPD-3NM devices? Include this information.

 (MI) P6, Structure of micropixel avalanche photodiodes: How stable is the described process? Which is the success7dfailure ration in manufacturing the devices?

 (MI) P6, Structure of micropixel avalanche photodiodes: PDE is described more than once in the text. It should only be described at its first appearance. Check and correct.

 (MI) P7, Methods: are there any other works already published that report the characteristics of the MAPD-3NK? How well do your research findings agree with those?

 (MI) P7, Methods: same comment as for Figure 1.

 (MI) P7, Methods: some more details regarding the experimental implementations are required. It will be helpful to include a sketch of the used set-up or some photos. Also, comment on the main difficulties that arose when doing these tests.

 (OP) P8, Results: Is there any disadvantage for the MAPD-3NM when compared to the MAPD-3NK?

 (OP) P10, Results: Is there any possibility for manufacturing different device sizes than 4x4 mm2? What are the limitations in device size?

 (OP) P10 Data availability: in “ *Advanced photodetector for hybrid PET-MRI systems, https://doi.org/10.17605/OSF.IO/5ZGU2*” the device area is 9 square mm but, in your submitted work, you mentioned 4 square mm. Please, comment on this difference and how it affects your results.

 (MI) P10-, References: Check that all references include all relevant information such as Authors, Volume, Page number…

 Thanks for considering my comments.

Is the study design appropriate and does the work have academic merit?

Yes

Is the work clearly and accurately presented and does it cite the current literature?

Partly

If applicable, is the statistical analysis and its interpretation appropriate?

Not applicable

Are all the source data underlying the results available to ensure full reproducibility?

Yes

Are the conclusions drawn adequately supported by the results?

Yes

Are sufficient details of methods and analysis provided to allow replication by others?

Partly

Reviewer Expertise:

PET instrumentation; SiPM technology;

I confirm that I have read this submission and believe that I have an appropriate level of expertise to confirm that it is of an acceptable scientific standard, however I have significant reservations, as outlined above.
